# Latent profile classification of cognitive function and its influencing factors in elderly patients with chronic pain after stroke

**DOI:** 10.3389/fnins.2026.1940893

**Published:** 2026-09-09

**Authors:** Jinglei Zhou, Lei Bo

**Affiliations:** Department of Neurology, Nanjing First Hospital, Nanjing Medical University, Nanjing, Jiangsu, China

**Keywords:** cognitive function, elderly, Latent Profile Analysis, post-stroke pain, risk factors

## Abstract

**Objectives:**

To identify the latent profile classification of cognitive function in elderly patients with chronic pain after stroke and explore the associated factors for patients in different categories.

**Methods:**

Elderly patients with chronic pain after stroke hospitalized in the Department of Neurology of a hospital in Nanjing from August 2025 to January 2026 were selected as research subjects. Data were collected using a General Information Questionnaire, the Montreal Cognitive Assessment (MoCA), Hamilton Anxiety Scale (HAMA), Center for Epidemiological Studies Depression Scale (CES-D), Pain Catastrophizing Scale (PCS), and Elderly Social Participation Scale. Latent Profile Analysis (LPA) was used for patient classification, and multivariate logistic regression analysis was performed to identify predictive factors for different groups (*P* < 0.05).

**Results:**

Patients’ cognitive function was classified into three latent profiles: High Cognitive Function-Low Abstraction Group, Moderate Cognitive Function-Low Orientation Group, and Low Cognitive Function Group. Logistic regression analysis showed that gender, pain type, pain intensity, anxiety, and depression were significant influencing factors of cognitive function across different categories (*P* < 0.05).

**Conclusion:**

There is significant population heterogeneity in the cognitive function of elderly patients with chronic pain after stroke. Medical staff should implement precise interventions for patients in different categories to delay cognitive decline and improve their quality of life.

## 1 Introduction

With the increasing aging of the population and the growing prevalence of risk factors for cerebrovascular diseases in China, the disease burden of stroke is showing an explosive growth trend (Tu et al., 2023). “Stroke” refers to a group of diseases caused by the sudden rupture of cerebral blood vessels or vascular obstruction that prevents blood flow to the brain, resulting in brain tissue damage. Globally, more than 12 million people suffer from stroke each year. In addition to disabilities caused by direct stroke-related injuries (such as motor dysfunction, speech disorders, and sensory disorders), over 50% of survivors experience chronic pain within weeks to months after the onset and continue to be afflicted by it (Feigin et al., 2022). Post-stroke pain includes headache, central pain, shoulder pain, and joint pain caused by spasticity and muscle weakness. Such patients are often accompanied by emotional disorders such as anxiety and depression, which are frequently undertreated, leading to reduced quality of life and a heavy social burden.

Furthermore, chronic pain is often comorbid with cognitive impairment. Epidemiological analyses of community residents and pain clinic patients estimate that at least 50% of chronic pain patients have concurrent cognitive impairment (Chen J. et al., 2023). Elderly stroke patients constitute a unique population with significant heterogeneity (Delpont et al., 2018). The cognitive function of elderly patients with chronic pain after stroke is influenced by multiple factors, which may vary across different subgroups. However, traditional research methods often struggle to capture these heterogeneous characteristics, potentially leading to suboptimal outcomes in disease management. Latent Profile Analysis (LPA) is an advanced analytical method that focuses on individual differences and uses latent classes to reveal relationships between observed variables.

In summary, this study employs LPA to accurately characterize the latent profiles of cognitive function in elderly patients with chronic pain after stroke, identify early predictors for patients in different categories, and clarify modifiable factors contributing to cognitive decline. The aim is to facilitate early detection of individuals at risk of cognitive impairment, delay cognitive decline, and improve patients’ quality of life.

## 2 Objects and methods

### 2.1 Study objects

Using convenience sampling, elderly patients with chronic pain after stroke hospitalized in the Department of Neurology of a hospital in Nanjing from August 2025 to January 2026 were enrolled.

Inclusion criteria: (1) Aged ≥ 60 years; (2) Diagnosed with stroke by cranial CT or MRI, consistent with the diagnostic criteria for stroke established at the 4th National Cerebrovascular Disease Conference in 1995; (3) Meeting the diagnostic criteria for chronic pain defined by the International Association for the Study of Pain (IASP) (Cohen et al., 2021), with pain duration ≥ 3 months and a Numerical Rating Scale (NRS) score > 1, all assessments were conducted concurrently at the time of enrollment; (4) Providing informed consent and agreeing to participate.

Exclusion criteria: (1) Pre-existing chronic pain before stroke; (2) Severe physical illness with inability to perform activities of daily living; (3) Visual or auditory dysfunction; (4) Pain caused by surgery or other accidental trauma.

### 2.2 Sample size calculation

This study primarily used multiple regression analysis, requiring a sample size at least 10 times the number of independent variables (Yan et al., 2017). A total of 35 independent variables were included in the regression model. Considering a 20% rate of invalid questionnaires, the minimum sample size was calculated as (35 × 10)/(1–0.2) = 438 cases. This study was conducted in accordance with the Declaration of Helsinki and was approved by the Ethics Committee of Nanjing First Hospital (the Institutional Review Board), approval number: KY20250811-KS-02.

### 2.3 Research tools

#### 2.3.1 General information questionnaire

Self-designed by the research team based on the study objectives, including age, gender, educational level, marital status, place of residence, comorbidities, smoking status, drinking status, duration of chronic pain, pain type, long-term use of analgesics (continuous medication for >2 weeks or intermittent medication for 1–3 days for >1 month), and pain intensity.

#### 2.3.2 Montreal Cognitive Assessment (MoCA)

Developed by Canadian scholars Nasreddine et al. (2019), this scale is a rapid screening tool for mild cognitive impairment. The version revised by Koski (2013) in 2006 was used, which includes seven cognitive domains: visuospatial/executive function (0–5 points), naming (0–3 points), attention (0–6 points), language (0–3 points), abstract thinking (0–2 points), delayed recall (0–5 points), and orientation (0–6 points). A score ≥ 26 indicates normal cognitive function, with higher scores reflecting better cognitive performance. The Cronbach’s α coefficient of the scale in this study was 0.734.

#### 2.3.3 Hamilton Anxiety Scale (HAMA)

Compiled by Hamilton (1959) and used by Huang et al. (2020) to assess anxiety severity in patients with cognitive impairment, this scale includes two dimensions (somatic anxiety: seven items; psychic anxiety: seven items) with 14 total items. A Likert five-point scoring system is used: “None” = 0 points, “Mild” = 1 point, “Moderate” = 2 points, “Severe” = 3 points, “Extremely severe” = 4 points. A total score ≥ 7 indicates possible anxiety, with higher scores indicating more severe anxiety. The Cronbach’s α coefficient was 0.860 in the original study and 0.887 in this study.

#### 2.3.4 Center for Epidemiological Studies Depression Scale (CES-D)

This scale assesses the frequency of depressive symptoms in the past week, including four dimensions (depressive mood, positive mood, somatic symptoms/retardation, and interpersonal relationships) and 20 items. Scoring criteria: <1 day = “None or almost none” (0 points), 1–2 days = “Rarely” (one point), 3–4 days = “Often” (two points), 5–7 days = “Almost always” (three points). Items 4, 8, 12, and 16 are reverse-scored. Total scores range from 0 to 60, with a score > 10 indicating depressive symptoms (Zhang et al., 2011).

#### 2.3.5 Pain Catastrophizing Scale (PCS)

Developed by Sullivan et al. (1995) to measure pain catastrophizing levels, this scale includes three dimensions (rumination: 4 items; magnification: 3 items; helplessness: 6 items) with 13 total items. A Likert fibe-point scoring system is used (0 = Never, 1 = Slightly, 2 = Moderately, 3 = Very, 4 = Always), with total scores ranging from 0 to 52. Higher scores indicate higher levels of pain catastrophizing, and a score ≥ 38 indicates a tendency toward pain catastrophizing. The Cronbach’s α coefficient was 0.927.

#### 2.3.6 Elderly Social Participation Scale

Compiled by Lu et al. (2022), this scale includes six dimensions (family activities, leisure activities, visits with relatives/friends, group cultural/recreational activities, voluntary public welfare activities, and online participation) with 22 items. A Likert five-point scoring system is used (1 = Never, 5 = Always), with higher scores indicating higher levels of social participation. The Cronbach’s α coefficient was 0.86.

### 2.4 Data collection methods

Research team members were trained before data collection to ensure familiarity with questionnaire content and administration procedures. Face-to-face interviews were conducted to collect information from elderly patients; informants were consulted if patients could not accurately answer questions. Questionnaires were collected on-site and checked for missing items. A total of 499 questionnaires were distributed, 49 invalid questionnaires were excluded, and 450 valid questionnaires were obtained, with an effective recovery rate of 90.2%.

### 2.5 Statistical methods

SPSS 26.0 and Mplus 8.3 software were used for statistical analysis, with a two-tailed test (*P* < 0.05) considered statistically significant. Baseline data were described using mean ± standard deviation, median (interquartile range), frequency, and percentage. LPA was used to identify latent profiles of cognitive function based on MoCA domain scores. The optimal model was selected by sequentially increasing the number of latent classes, considering model fit indices (AIC, BIC, aBIC) and comparative indices (Entropy, LMR, BLRT). LPA was conducted using Mplus 8.3 with 500 random starts and 50 final-stage optimizations. The optimal 3-class solution was replicated across multiple sets of random starts, and no estimation problems (e.g., non-positive definite covariance matrices, boundary estimates) were detected.

Univariate analysis was used to screen statistically significant independent variables, and multinomial logistic regression analysis was performed to identify predictive factors for different cognitive function profiles.

## 3 Results

### 3.1 Current status of patients’ cognitive function

The average total MoCA score of the 450 patients with chronic pain after stroke was 20.09 ± 5.14. The average scores for each domain were: visuospatial/executive function (3.06 ± 1.75), naming (1.95 ± 1.16), attention (3.84 ± 1.94), language (1.99 ± 0.91), abstract thinking (1.33 ± 0.75), delayed recall (3.38 ± 1.39), and orientation (4.55 ± 1.96).

### 3.2 Latent profiles of cognitive function

To explore the latent profiles of cognitive function, four potential models (1–4 classes) were tested for fit (Table 1). The AIC and BIC values decreased progressively with an increasing number of classes, indicating better model fit. The Entropy value for the 3-class model was 0.961, suggesting excellent classification accuracy. Additionally, the LMR and BLRT results for the 2, 3, and 4-class solutions were significant (*P* < 0.05), confirming that increasing the number of classes significantly improved model fit. Although the 4-class model had lower information criteria, the 3-class model was selected as the optimal model based on its superior interpretability, parsimony, and clinically meaningful class probabilities (23.2%, 66.5%, 10.3%), avoiding potential overfitting.

**TABLE 1:** Fit indices for Latent Profile Analysis models of cognitive function.

Model	K	AIC	BIC	aBIC	Entropy	LMR *p*-value	BLRT *p*-value	Class probabilities (%)
1-class	14	10730.625	10788.155	10743.724	–	–	–	100
2-class	22	10403.638	10494.041	10424.221	0.947	0.0000	0.0000	28.1/71.9
3-class	30	10248.734	10372.011	10276.803	0.961	0.0004	0.0000	23.2/66.5/10.3
4-class	38	10124.027	10280.178	10159.581	0.972	0.0000	0.0000	8.6/17.0/10.2/64.2

K, number of free parameters; AIC, Akaike Information Criterion; BIC, Bayesian Information Criterion; aBIC, sample-size adjusted BIC; LMR, Lo-Mendell-Rubin test; BLRT, Bootstrap Likelihood Ratio Test.

### 3.3 Nomenclature of cognitive function profiles

**FIGURE 1:**
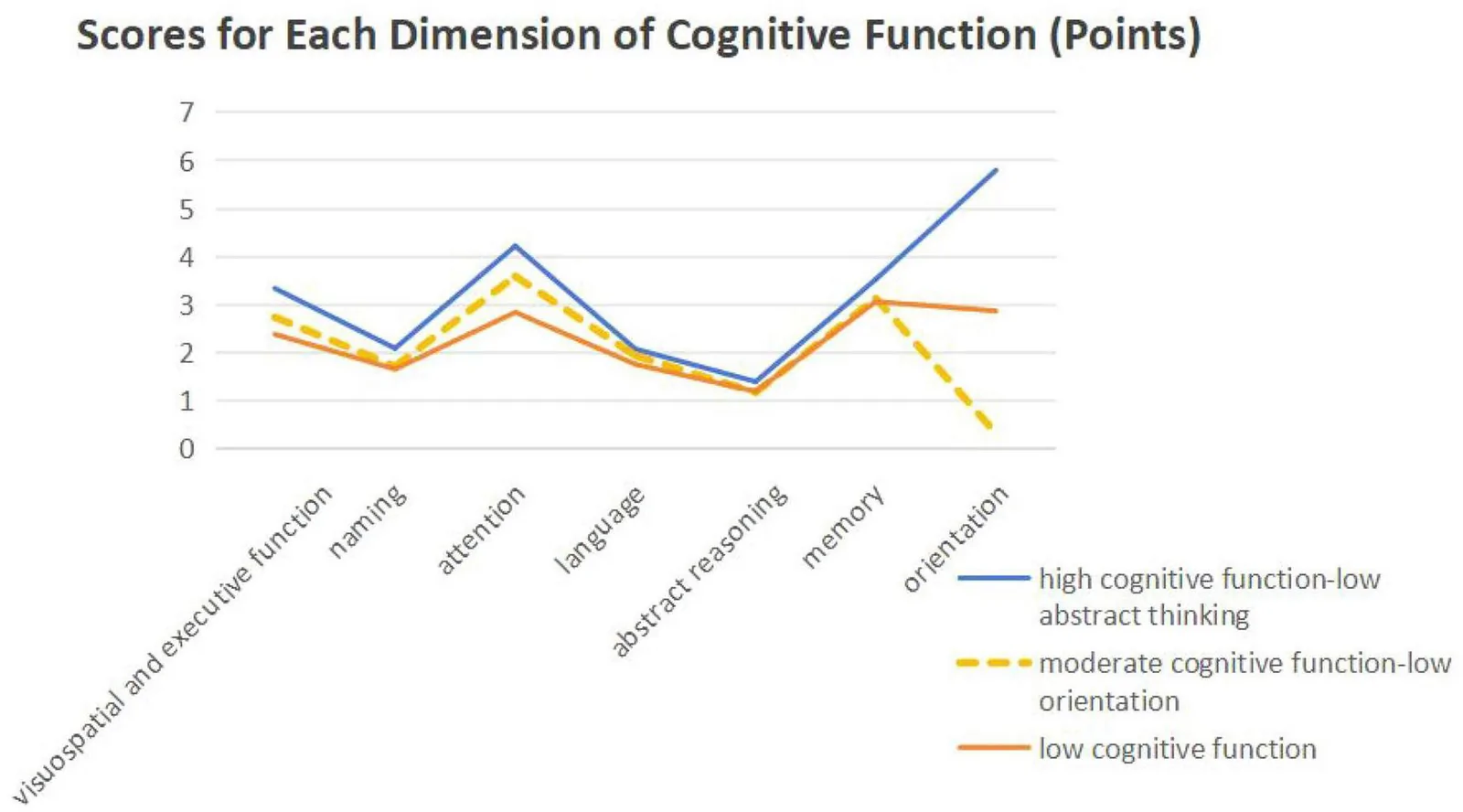
Radar chart of cognitive domain scores for the three latent profiles.

Based on the patterns of domain-specific scores, the three latent classes were identified and named as follows (Figure 1): Class 1 (High Cognitive Function-Low Abstraction Group, *n* = 299, 66.4%) demonstrated the highest scores across most cognitive domains but exhibited a relative weakness in abstract thinking; Class 2 (Moderate Cognitive Function-Low Orientation Group, *n* = 105, 23.3%) showed moderate scores across all domains with a distinct deficit specifically in the orientation domain; and Class 3 (Low Cognitive Function Group, *n* = 46, 10.2%) exhibited significantly lower scores across all cognitive domains, indicating global cognitive impairment.

### 3.4 Univariate analysis of latent profiles

Univariate analysis revealed that gender, comorbidities, pain type, pain intensity, anxiety, depression, and pain catastrophizing were significantly associated with the latent cognitive function profiles (*P* < 0.05). Detailed results are presented in Table 2.

**TABLE 2:** Univariate analysis of factors associated with latent cognitive function profiles (*n* = 450).

Variables	C1 (high-low abstraction) (*n* = 299)	C2 (moderate-low orientation) (*n* = 105)	C3 (low cognitive function) (*n* = 46)	χ ^2^ or H	*P*-value
Gender				23.271	0.000*
Male	177 (59.0%)	83 (27.7%)	40 (13.3%)	–	–
Female	122 (81.3%)	22 (14.7%)	6 (4.0%)	–	–
Comorbidities				6.535	0.038*
No	50 (80.6%)	8 (12.9%)	4 (6.5%)	–	–
Yes	249 (64.2%)	97 (25.0%)	42 (10.8%)	–	–
Pain type				36.205	0.000*
Central pain	130 (82.8%)	20 (12.7%)	7 (4.5%)	–	–
Shoulder pain	94 (59.5%)	45 (28.5%)	19 (12.0%)	–	–
Complex regional pain syndrome	54 (52.4%)	30 (29.1%)	19 (18.4%)	–	–
Headache	21 (65.6%)	10 (31.3%)	1 (3.1%)	–	–
Pain intensity				13.560	0.009*
Mild	157 (65.7%)	52 (21.8%)	30 (12.6%)	–	–
Moderate	75 (59.5%)	41 (32.5%)	10 (7.9%)	–	–
Severe	67 (78.8%)	12 (14.1%)	6 (7.1%)	–	–
Anxiety				39.717	0.000*
Mild	99 (85.3%)	16 (13.8%)	1 (0.9%)	–	–
Moderate	103 (52.6%)	61 (31.1%)	32 (16.3%)	–	–
Severe	70 (70.0%)	20 (20.0%)	10 (10.0%)	–	–
Extremely severe	27 (71.1%)	8 (21.1%)	3 (7.9%)	–	–
Depression				15.701	0.000*
No	249 (71.1%)	71 (20.3%)	30 (8.6%)	–	–
Yes	50 (50.0%)	34 (34.0%)	16 (16.0%)	–	–
Pain catastrophizing				9.144	0.010*
No tendency	240 (69.8%)	69 (20.1%)	35 (10.2%)	–	–
With tendency	59 (55.7%)	36 (34.0%)	11 (10.4%)	–	–

Data are presented as *n* (%). C1, High Cognitive Function-Low Abstraction Group, C2, Moderate Cognitive Function-Low Orientation Group, C3, Low Cognitive Function Group. The Kruskal-Wallis H test was used for social participation (data not shown, *P* = 0.425). The χ^2^ test was used for other categorical variables. *P < 0.05 indicates statistical significance.

### 3.5 Multivariate logistic regression analysis of latent profiles

Statistically significant variables from the univariate analysis were included in a multinomial logistic regression model, with the “High Cognitive Function-Low Abstraction Group” (C1) serving as the reference category (Table 3).

**TABLE 3:** Variable assignment for multivariate logistic regression.

Variable type	Variable name	Assignment
Dependent	Cognitive function profile	1 = high-low abstraction (ref); 2 = moderate-low orientation; 3 = low cognitive function
Independent	Gender	0 = male; 1 = female (ref)
	Comorbidities	0 = no; 1 = yes (ref)
	Pain type	0 = central pain; 1 = shoulder pain; 2 = complex regional pain syndrome; 3 = headache (ref)
	Pain intensity	0 = mild; 1 = moderate; 2 = severe (ref)
	Anxiety	0 = mild; 1 = moderate; 2 = severe; 3 = extremely severe (ref)
	Depression	0 = no; 1 = yes (ref)
	Pain catastrophizing	0 = no tendency; 1 = with tendency (ref)

The regression analysis revealed several significant predictors, as detailed in Table 4. Compared with the High Cognitive Function-Low Abstraction Group (C1), male patients were significantly more likely to be classified into the Moderate Cognitive Function-Low Orientation Group (C2) (OR = 4.006, 95% CI: 1.553–10.330, *P* = 0.004), whereas the absence of depression (OR = 0.402, 95% CI: 0.224–0.721, *P* = 0.002) and mild anxiety (OR = 0.092, 95% CI: 0.009–0.989, *P* = 0.049) were associated with lower odds of belonging to this group. Furthermore, when comparing the Low Cognitive Function Group (C3) with the reference group (C1), male patients (OR = 2.127, 95% CI: 1.197–3.779, *P* = 0.010), as well as those with mild anxiety (OR = 2.294, 95% CI: 1.080–4.873, *P* = 0.031) or moderate anxiety (OR = 3.612, 95% CI: 1.627–8.014, *P* = 0.002), were more likely to be in the C3 group. Conversely, patients with central pain (OR = 0.304, 95% CI: 0.113–0.818, *P* = 0.018) and those without depression (OR = 0.327, 95% CI: 0.147–0.726, *P* = 0.006) were less likely to be classified into this lowest cognitive function group.

**TABLE 4:** Multivariate logistic regression analysis of cognitive function profiles.

Group (vs. high-low abstraction)	Variables	B	Std. error	Wald	*P*-value	OR	95% CI for OR
Moderate-low orientation	Intercept	−2.430	1.775	1.874	0.171	–	–
	Gender (male)	1.388	0.483	8.243	0.004	4.006	1.553–10.330
	Anxiety (mild)	−2.387	1.212	3.877	0.049	0.092	0.009–0.989
	Depression (no)	−0.912	0.298	9.351	0.002	0.402	0.224–0.721
Low cognitive function	Intercept	−0.258	0.429	0.361	0.548	–	–
	Gender (male)	0.755	0.293	6.624	0.010	2.127	1.197–3.779
	Pain type (central pain)	−1.192	0.506	5.560	0.018	0.304	0.113–0.818
	Anxiety (mild)	0.830	0.384	4.663	0.031	2.294	1.080–4.873
	Anxiety (moderate)	1.284	0.407	9.970	0.002	3.612	1.627–8.014
	Depression (no)	−1.117	0.407	7.542	0.006	0.327	0.147–0.726

Reference group: High Cognitive Function-Low Abstraction Group. Only significant results (*P* < 0.05) are shown for brevity. The model was adjusted for comorbidities, pain intensity, and pain catastrophizing, which were not significant in the final model. Gender reference, female; pain type reference, headache; anxiety reference, extremely severe; depression reference, yes.

## 4 Discussion

### 4.1 Current status of cognitive function in elderly patients with chronic pain after stroke

This study identified three distinct latent profiles of cognitive function in elderly patients with chronic pain after stroke, with an overall average MoCA score of 20.09 ± 5.14, indicating a moderately low level of cognitive function. This finding is consistent with previous research (Niu et al., 2025) and supports the established link between chronic pain and impairments in multiple cognitive domains, including memory, attention, and executive function (Berryman et al., 2014). A potential mechanism is the “bottom-up” hypothesis, where persistent pain signals “occupy” cerebral cognitive resources, leading to functional and microstructural changes in brain regions like the prefrontal cortex and hippocampus (Chen J. et al., 2023). Given the high prevalence of stroke in China, addressing the cognitive sequelae of post-stroke chronic pain is crucial for reducing the associated family and societal burden.

### 4.2 Heterogeneity of latent cognitive function profiles

The identification of three distinct profiles confirms the heterogeneity of cognitive function among elderly patients with chronic pain after stroke. The High Cognitive Function-Low Abstraction Group (66.4%) comprised patients with relatively preserved cognitive function, exhibiting only a specific weakness in abstract thinking, which may serve as an early marker of frontal lobe dysfunction associated with chronic pain (Berryman et al., 2014). The overall cognitive stability observed in this group may be attributed to better health literacy and disease self-management capabilities (Claassen et al., 2021), suggesting that continued health education to maintain physical and mental stability is paramount for these individuals. In contrast, the Moderate Cognitive Function-Low Orientation Group (23.3%) demonstrated moderate overall cognition with a pronounced deficit in orientation. Chronic pain can disrupt attention and orientation through mechanisms such as sleep disturbance and reduced social interaction (Terassi et al., 2021; Zhu et al., 2025), positioning this group in a potential transitional state where targeted interventions, including cognitive training focused on time and place orientation, could be particularly beneficial. The Low Cognitive Function Group (10.2%) exhibited severe, global cognitive impairment, likely resulting from a synergistic interaction among stroke pathology, aging, chronic pain, and multiple comorbidities (Zhu et al., 2025). As the highest priority for clinical intervention, these patients require comprehensive geriatric assessments and integrated care models that combine pharmacological and non-pharmacological approaches-such as cognitive stimulation and physical activity-to prevent further decline and progression to dementia.

### 4.3 Influencing factors of cognitive function profiles

#### 4.3.1 Gender

Our study found that male patients had significantly higher odds of being in both the moderate and low cognitive function groups compared to females. While females often report higher pain sensitivity (Luo et al., 2026), they may also employ more effective emotional coping strategies and be more willing to seek social support, which could be protective against cognitive decline (Calvó-Perxas et al., 2016). Males, conversely, might underreport pain and emotional distress, leading to undertreatment and a greater cumulative negative impact on cognition. These findings underscore the need for gender-sensitive approaches in clinical practice, encouraging open communication about pain and emotional health, particularly with male patients.

#### 4.3.2 Pain type

Contrary to some previous reports (Mohanan et al., 2023), central post-stroke pain (CPSP) appeared as a protective factor against being in the Low Cognitive Function Group. One possible explanation is that CPSP, often being severe and distinct, is more readily recognized and treated, potentially leading to better pain management and reduced cognitive burden. In contrast, complex regional pain syndrome, which is often more challenging to treat, was associated with worse cognitive outcomes in the univariate analysis. However, it is critical to note that a large proportion of CPSP patients still receive inadequate treatment (Jiang et al., 2025; Mohanan et al., 2023). This finding highlights the importance of improving clinicians’ ability to identify and effectively manage all types of post-stroke pain, especially refractory conditions.

#### 4.3.3 Pain intensity, anxiety, and depression

Our results confirm the strong interplay between pain, negative emotions, and cognition. Anxiety emerged as a powerful risk factor, with mild and moderate anxiety significantly increasing the likelihood of belonging to the Low Cognitive Function Group. Chronic pain is known to increase the risk of anxiety and depression by 2.5–4.1 times (Chen J. A. et al., 2023), and shared neurobiological mechanisms, such as neuroinflammation and maladaptive neuroplasticity, underpin this comorbidity (Chen J. A. et al., 2023). Furthermore, depression was a significant predictor, consistent with research suggesting pain intensity may indirectly affect cognition through its impact on mood (Shi et al., 2025). These findings strongly advocate for integrated treatment models that address pain, anxiety, and depression concurrently through approaches like cognitive-behavioral therapy and appropriate pharmacotherapy to optimize cognitive outcomes.

## 5 Conclusion

This study identified three latent profiles of cognitive function in elderly patients with chronic pain after stroke: High Cognitive Function-Low Abstraction (66.4%), Moderate Cognitive Function-Low Orientation (23.3%), and Low Cognitive Function (10.2%). These findings confirm the heterogeneity of cognitive function in this population and demonstrate the value of person-centered approaches like LPA over traditional variable-centered methods. Gender, pain type, pain intensity, anxiety, and depression were significant predictors of profile membership.

Clinically, healthcare providers should recognize this heterogeneity and tailor interventions accordingly. The High Cognitive Function-Low Abstraction Group may only require continued health education and monitoring. The Moderate Cognitive Function-Low Orientation Group could benefit from targeted orientation training combined with interventions addressing sleep and social engagement. The Low Cognitive Function Group needs comprehensive integrated care combining pharmacological and non-pharmacological approaches to prevent further decline.

This study has several limitations. The single-center convenience sampling and cross-sectional design limit generalizability and preclude causal inferences. In addition, we were unable to assess potential confounders such as medications, nutritional status, and physical activity. Regarding measurement, the Elderly Social Participation Scale used in this study has not been formally validated in stroke populations, although it showed good internal consistency in our sample. Future multi-center longitudinal studies are needed to validate these findings, track cognitive trajectories, and identify additional modifiable risk factors. Despite these limitations, this study provides valuable insights into cognitive heterogeneity in this vulnerable population and establishes a foundation for developing personalized interventions.

## Data Availability

The raw data supporting the conclusions of this article will be made available by the authors, without undue reservation.
